# Vigorous-Intensity Physical Activities Are Associated with High Brown Adipose Tissue Density in Humans

**DOI:** 10.3390/ijerph17082796

**Published:** 2020-04-18

**Authors:** Riki Tanaka, Sayuri Fuse, Miyuki Kuroiwa, Shiho Amagasa, Tasuki Endo, Akira Ando, Ryotaro Kime, Yuko Kurosawa, Takafumi Hamaoka

**Affiliations:** 1 Department of Sports Medicine for Health Promotion, Tokyo Medical University, Tokyo 160-8402, Japan; tmur.tanaka@gmail.com (R.T.); fuse@tokyo-med.ac.jp (S.F.); mkuroiwa@tokyo-med.ac.jp (M.K.); endo@tokyo-med.ac.jp (T.E.); kime@tokyo-med.ac.jp (R.K.); kurosawa@tokyo-med.ac.jp (Y.K.); 2 Department of Preventive Medicine and Public Health, Tokyo Medical University, Tokyo 160-8402, Japan; shiho.ama@gmail.com; 3 Japan Institute of Sports Sciences, Tokyo 115-0056, Japan; aand2.x2@gmail.com

**Keywords:** brown adipose tissue (BAT), vigorous-intensity physical activities (VPA), near-infrared time-resolved spectroscopy (NIR_TRS_), exercise, sympathetic nervous system (SNS)

## Abstract

Brown adipose tissue (BAT) plays a role in adaptive thermogenesis in response to cold environments and dietary intake via sympathetic nervous system (SNS) activation. It is unclear whether physical activity increases BAT density (BAT-d). Two-hundred ninety-eight participants (age: 41.2 ± 12.1 (mean ± standard deviation), height: 163.6 ± 8.3 cm, weight: 60.2 ± 11.0 kg, body mass index (BMI): 22.4 ± 3.0 kg/m^2^, body fat percentage: 25.4 ± 7.5%) without smoking habits were categorized based on their physical activity levels (a group performing physical activities including walking and moderate physical activity (WM) and a group performing WM + vigorous-intensity physical activities (VWM)). We measured the total hemoglobin concentration ([Total-Hb]) in the supraclavicular region, an index of BAT-d, and anthropometric parameters. [Total-Hb] was significantly higher in VWM than WM for all participant groups presumably owing to SNS activation during vigorous-intensity physical activities, and unrelated to the amount of total physical activity levels. Furthermore, multiple regression analysis revealed that BAT-d was related to visceral fat area and VWM in men and related to body fat percentage in women. We conclude that vigorous-intensity physical activities are associated with high BAT-d in humans, especially in men.

## 1. Introduction

Human brown adipose tissue (BAT) is present in the supraclavicular, cervical, and paravertebral regions [1,2] and plays a role in adaptive thermogenesis in response to cold environments and dietary intake [3,4,5,6,7,8,9], thereby increasing systemic energy output [10]. In particular, the mechanism for cold-induced thermogenesis in BAT is achieved through activation of the sympathetic nervous system (SNS) via the transient receptor potential (TRP) channel in the skin [11,12,13,14,15,16]. BAT is prominent in newborns and infants, deteriorating with growth, and disappearing with aging [17]. In addition to aging and cold stimulation, specific drugs, dietary ingredients, circadian rhythms, and exercise have been reported as factors affecting BAT activity [3,4,5,18,19,20,21,22,23,24,25]. However, many aspects are unclear regarding the relationship between exercise and BAT [19]. In animal models investigating the relationship between BAT and exercise, endurance exercise training elicited increased BAT activity and white adipose tissue (WAT) browning [26,27,28]. In human studies, men and women who performed endurance exercises in cross-sectional studies have reported significantly lower cold-induced ^18^F-fluorodeoxyglucose (^18^F-FDG) uptake, an indicator of BAT activity, than sedentary men and non-athlete women [23,24]. In contrast, it has been reported that ^18^F-FDG uptake does not change after two weeks of high intensity interval training or moderate training in humans [25].

BAT has been evaluated in the supraclavicular region using ^18^F-FDG–positron emission tomography (PET) with computed tomography (CT) (^18^FDG-PET/CT) in humans [29,30,31]. This method has several limitations such as radiation exposure, considerable instrumentation costs, and acute cold exposure [32,33,34], which make repeated evaluation of BAT in healthy individuals difficult. Therefore, a noninvasive, simple method, which does not require exposure to cold or radiation, is desirable [35]. Near-infrared time-resolved spectroscopy (NIR_TRS_) can be used to measure total hemoglobin concentration ([Total-Hb]) and oxygenation in biological tissues [36,37,38]. The abundant capillaries of BAT allow NIR_TRS_ to distinguish the characteristics of BAT from WAT [36,37,38]. BAT evaluation using NIR_TRS_ does not require cold exposure or radiation exposure, so it can be evaluated safely and non-invasively [39]. [Total-Hb] evaluated by NIR_TRS_ positively correlated with BAT parameters evaluated by ^18^FDG–PET/CT only in the supraclavicular region, which is the location of BAT [39]. Longitudinal studies revealed that an increase in BAT activity induced by repeated ingestion of thermogenic food ingredients can be detected by an increase in [Total-Hb] [40]. Collectively, [Total-Hb] in the supraclavicular region evaluated by NIR_TRS_ is expected to be suitable for evaluating BAT density (BAT-d) and equivalent to the determination of BAT activity or BAT volume by ^18^FDG-PET/CT using cold exposure [39,40,41,42].

A question arises whether exercise can modulate BAT-d, and if so, which intensity can effectively increase BAT-d in humans. SNS plays an important role in activating BAT and increasing BAT volume [11,12,13,14,15,16]. The response of SNS during exercise is reported to increase from around 50–70% of the maximal oxygen uptake (VO_2max_) [43,44,45,46,47]. However, previous exercise studies did not focus on SNS, which makes previous research results regarding the correlation of exercise with BAT unclear [27,48,49,50,51,52,53,54,55]. We hypothesized that vigorous-intensity physical activity (VPA) activates SNS, yielding an increase in BAT-d in the supraclavicular region. The purpose of this study was to clarify the effect of VPA on BAT-d.

## 2. Materials and Methods

### 2.1. Participants and Study Design

The study was conducted using a cross-sectional design. Participants were recruited using advertisements or direct contact. Among 319 healthy men and women, aged 20 years or older, who participated in the study from December to March, 298 participants without smoking habits were categorized based on their physical activity levels. The participants responded to a questionnaire and were evaluated in the laboratory for BAT-d, body composition, blood pressure, and heart rate. This study was conducted in accordance with the Declaration of Helsinki, and the protocol was approved by the Tokyo Medical University Medical Ethics Review Board (approval number: SH3957). All participants had given a written consent before participating in the study.

### 2.2. Brown Adipose Tissue Density

BAT-d was evaluated by NIR_TRS_ (Hamamatsu Photonics K.K., Hamamatsu, Japan) according to previous studies [39,56]. A probe with an optode distance of 3 cm was used. The light could reach a mean depth of 2 cm [57], a depth at which BAT is expected [58]. [Total-Hb] in the supraclavicular region, an index of BAT-d, was calculated as the sum of oxygenated Hb and deoxygenated Hb. Reduced scattering coefficient (μ’_s_), which is one of the tissue optical characteristics, was also measured. The [Total-Hb] in the supraclavicular region is adjusted according to the thickness of the subcutaneous adipose tissue layer (1.00 ± 0.48 mm) [59]. NIR_TRS_ data were extracted every 10 seconds. The coefficient of variation within an individual when evaluated repeatedly is 4.9% [39].

### 2.3. Physical Activity Level

We evaluated the total amount of physical activity using the international physical activity questionnaire (IPAQ, long version) during a representative week for the time of physical activity, time spent on each activity [walking (W), moderate-intensity physical activity (MPA), and VPA, and energy expenditure. IPAQ evaluates physical activity lasting longer than 10 minutes. The total amount of physical activity was calculated by integrating the duration of the physical activity (hours) and the physical activity intensity (METs). Based on the IPAQ analysis guidelines, we assigned 3.3 METs for W, 4.0 METs for MPA, and 8.0 METs for VPA [60]. According to the reference value for Japanese men by the Ministry of Health, Labor and Welfare, the level of 8 METs corresponds to approximately 80% VO_2max_ in participants of the average age in this study (41.3 years) [61]. Thus, the intensity of VPA categorized in this study is considered sufficient to activate SNS [47].

### 2.4. Measurement of Anthropometric and Circulatory Parameters

We measured body height, weight, body fat mass, body fat percentage, skeletal muscle mass, skeletal muscle percentage, waist circumference, and visceral fat area. Body weight, body fat mass, body fat percentage, and skeletal muscle mass were measured by bioelectric impedance (Inbody 720 Body Composition Analyzer; InBody Japan, Tokyo, Japan) [62,63]. The subcutaneous adipose thickness of the supraclavicular, deltoid, and abdominal regions was monitored using B-mode ultrasonography (Vscan Dual Probe; GE Vingmed Ultrasound AS, Horten, Norway). Waist circumference and visceral fat area were measured in an upright position using an impedance method (Bioelectrical impedance analysis EW-FA90; Panasonic, Osaka, Japan). Systolic blood pressure, diastolic blood pressure, and heart rate were measured using an automated sphygmomanometer (HEM-1025; Omron Healthcare, Kyoto, Japan). Body mass index (BMI) was calculated by weight (kg) per height squared (m^2^), and skeletal muscle percentage was calculated by skeletal muscle mass (kg) per body weight (kg).

### 2.5. Statistical Analysis

Twenty-one participants with smoking habits were excluded from the analysis. The remaining participants were classified according to whether they performed VPA with potential to increase SNS activity. All 232 participants (77 individuals who performed VWM and 155 persons who performed WM group) (men: 87; women: 145) were included in the analysis. The values are shown in the mean ± standard deviation. To determine the difference between the WM and VWM groups, an independent t-test was used. The Mann–Whitney test was used to analyze the energy expenditure, time of VPA, time of moderate physical activity, and time of physical activity related to walking according to the guidelines [60]. The BAT-d level, level of physical activity intensity (with or without vigorous activity), total amount of physical activity, and gender were analyzed by two-way analysis of variance. To evaluate factors correlating with BAT-d, we used stepwise multiple regression analysis with BAT-d as the independent variable and age, body fat percentage, visceral fat area, and with (1)/without (0) VPA, as the dependent variables.

All analyses were performed using the SPSS software (IBM SPSS Statistics 25 and/or 26, IBM Japan, Tokyo, Japan), and *p* < 0.05 was considered statistically significant.

## 3. Results

### 3.1. Participant Characteristics

**Figure 1:**
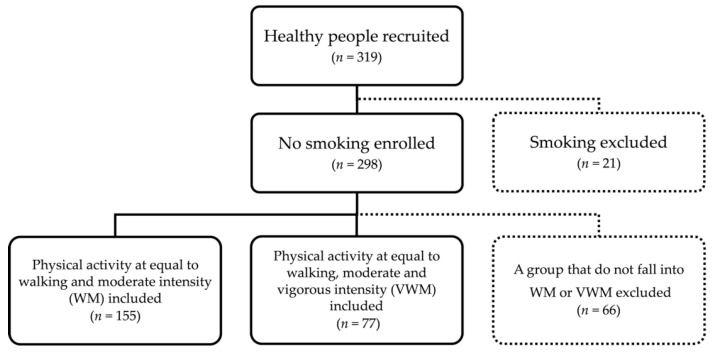
Inclusion/exclusion criteria for the participants. Two-hundred ninety-eight people without smoking habits were extracted. Among them, we analyzed 232 healthy men and women after excluding 66 participants who were not categorized in a group performing all physical activities (including walking, moderate physical activity, and vigorous-intensity physical activity) or a group performing some physical activities (including walking and moderate-intensity physical activity).

The participants of this study were 298 healthy non-smokers (age: 41.2 ± 12.1, height: 163.6 ± 8.3 cm, weight: 60.2 ± 11.0 kg, BMI: 22.4 ± 3.0 kg/m^2^, body fat percentage: 25.4 ± 7.5%). Figure 1 shows the inclusion/exclusion criteria. We analyzed 232 healthy men and women after excluding 66 individuals; the 232 participants were categorized into 2 groups: a group performing WM and a group performing VWM.

The μ’_s_ in the supraclavicular region was found to be 8.3 (7.3, 9.4) (medians (the first quartile, the third quartile)) cm^−1^ and (total-Hb), 65.1 (50.4, 85.0) μM. Compared to the WM group, the VWM group had a significantly higher [Total-Hb] in the supraclavicular region but similar height, weight, skeletal muscle mass, skeletal muscle percentage, lean body mass, systolic blood pressure, energy expenditure by physical activity, and the time of physical activity at equal to walking. However, the body fat mass and body fat percentage were significantly lower in the VWM group than in the WM group (Table 1).

In women, there was no significant difference in [Total-Hb] in the supraclavicular region between the VWM and WM groups. The skeletal muscle percentage and energy expenditure by physical activity were significantly higher in the VWM group compared to the WM group; only body fat mass was significantly lower in the VWM group (Table 2).

In men, compared with the WM group, the VWM group showed significantly higher [Total-Hb] in the supraclavicular region and increased skeletal muscle mass, skeletal muscle percentage, lean body mass, energy expenditure by physical activity, and the time of physical activity at equal to walking. Body fat mass and body fat percentage were significantly lower in the VWM group than in the WM group (Table 3).

### 3.2. Association between Vigorous-Intensity Physical Activity and [Total-Hb] in the Supraclavicular Region, an Index of Brown Adipose Tissue Density

**Figure 2:**
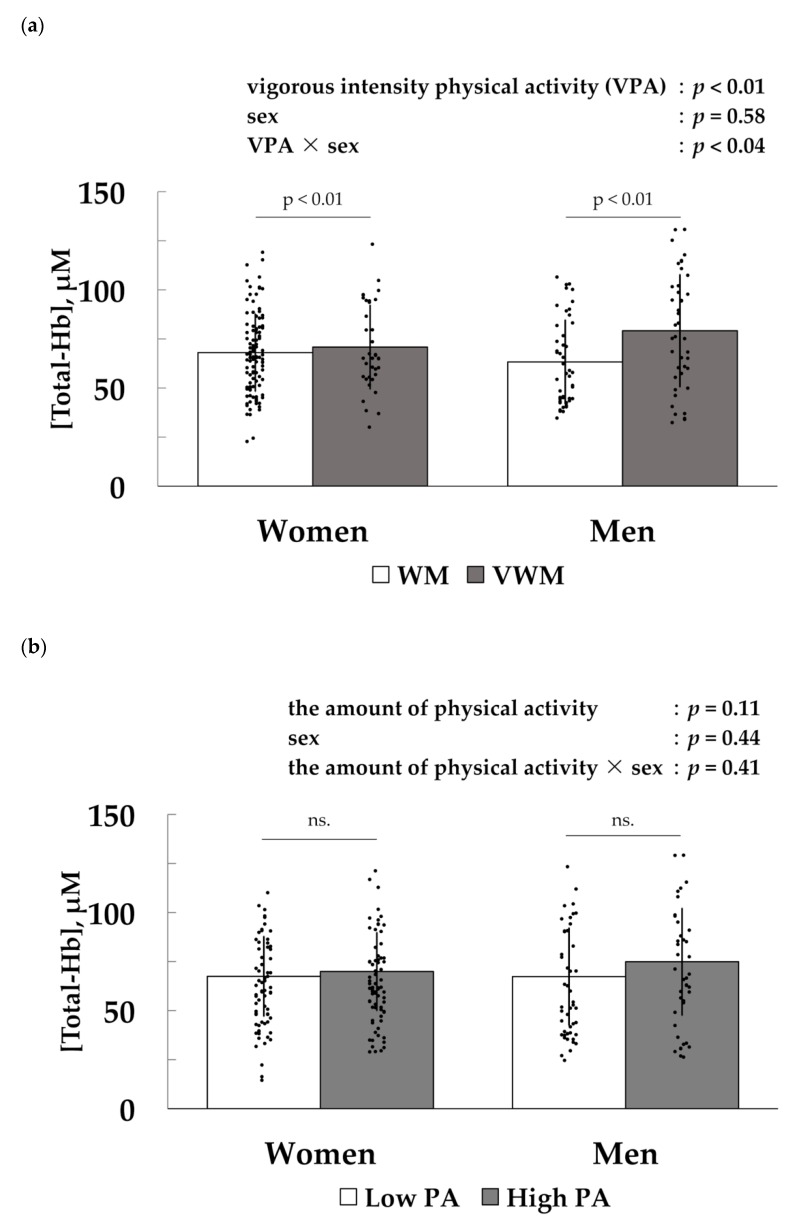
Comparison of [Total-Hb] in the supraclavicular region, an index of brown adipose tissue density (**a**) between a group performing all physical activities including walking, moderate physical activity, and vigorous-intensity physical activity (VWM) and a group performing physical activities including walking and moderate physical activity (WM); (**b**) between the higher physical activity (High PA) group and the lower physical activity (Low PA) group. Results are presented as the mean ± standard deviation. Two-way analysis of variance was performed to determine the significance of group and gender differences; ns., not significant.

Two-way analysis of variance showed a significant interaction with a main effect in the group (Figure 2a). There was no significant relationship between BAT-d and the time spent performing VPA and between BAT-d and the frequency each week performing VPA. There was no significant difference in the amount of total physical activity between groups (Figure 2b).

### 3.3. Factors Associated with [Total-Hb] in the Supraclavicular Region, an Index of Brown Adipose Tissue Density

In all participants, the body fat percentage and visceral fat area were significantly related to [Total-Hb]. In women, the body fat percentage was significantly related to [Total-Hb]. In men, visceral fat area and VPA were significantly related to [Total-Hb] (Table 4).

## 4. Discussion

In this study, participants, especially men, performing VPA, showed high BAT-d in the supraclavicular region. Information is sparse from human studies investigating the relationship between exercise and BAT; previous studies have focused on aerobic exercise [19,50], vigorous-intensity exercise [25], and muscle strength [64]. This study was the first to observe a relationship between VPA and BAT-d. Furthermore, multiple regression analysis of BAT-d as the independent variable and age, body adiposity, and VPA as the dependent variables revealed that BAT-d is related to visceral fat area and VPA only in men. The reason for this observation is not clear but may be related to androgens [65]. Differentiated association of BAT-d and fat distribution between men and women observed in the study may explained by an evidence that estrogens increase the sympathetic tone differentially to the adipose tissue depots favoring lipid accumulation in the subcutaneous fat in women and visceral fat deposition in men [66].

The mechanism of cold-induced BAT activation is well known [67,68,69,70]. When the TRP channel in the skin receives a cold stimulus, the generated afferent nerve impulse is transmitted to the dorsal horn of the spinal cord and further to the hypothalamic preoptic area (POA), the thermoregulatory center. When the nerve impulse is received by POA, disinhibition of the thermogenic neuron in the dorsomedial hypothalamus and sympathetic and somatic premotor neuron in the rostral medullary raphe region occurs to prevent hypothermia. Eventually, a nerve impulse from the excited SNS is transmitted to BAT via sympathetic preganglionic neurons (SPNs) located in the spinal intermediolateral nucleus (IML) of the spinal ventral horn (SVH). When released noradrenaline from the sympathetic nerve ends binds to β_3_ adrenergic receptors on brown adipocyte membranes, uncoupling protein 1 (UCP-1) on the inner mitochondrial membrane is activated, and BAT thermogenesis is induced [67,68,69,70].

It is postulated that exercise has an analogous mechanism to cold-induced enhancement of BAT via SNS activation. Exercise-induced mechanical and metabolic stimuli in the periphery are integrated in the circulatory center of the medulla oblongata via afferent fibers (group III, IV). The integrated information is transmitted to SPNs located in the SVH IML, which activate cardiac sympathetic nerves, muscle vasoconstrictor nerves, and presumably also BAT [71]. In humans, a 6-minute rowing ergometer exercise resulted in a 2-fold increase in the blood levels of the inflammatory cytokine IL-6, at rest [72]. Inflammatory cytokines such as IL-6 induce the expression of prostaglandin synthases such as cyclooxygenase-2 in cerebral vascular endothelial cells, leading to prostaglandin E2 (PGE2) production [73]. When PGE2 binds to the POA receptor EP3, disinhibition of the sympathetic nerve drive is suppressed, leading to an increase in SNS through a pathway similar to cold stimulation, which activates BAT thermogenesis [74].

In previous cross-sectional studies investigating the relationship between exercise and BAT, the ^18^F-FDG uptake in men and women performing endurance exercise was significantly lower than in sedentary men and non-athlete women [23,24]. On the other hand, two weeks of high intensity interval training and moderate training interventions did not alter ^18^F-FDG uptake [25]. A cross-sectional report on the relationship between muscle strength and BAT activity showed a positive correlation between grip strength and BAT activity [64]. The concentration of blood myokine, a marker for promoting WAT browning, has been reported to be higher in an 8-week resistance training group than in control and endurance training groups [75,76]. Moreover, 12 weeks of moderate- and vigorous-intensity bicycle training increased UCP-1 mRNA expression in human abdominal WAT [77]. From these previous studies, VPA and/or related physiological modifications were expected to affect BAT activity and WAT browning. Therefore, in this study, we investigated the relationship between VPA accompanied with increased SNS activity and BAT-d in the supraclavicular region. We found that men performing VPA showed high BAT-d in the supraclavicular region. The results of the association between BAT-d and VPA in men are consistent with a previous report that androgen has the potential to promote browning of WAT in animal models [65]. Men have larger muscle mass, which may lead enhanced myokine levels and an increase in BAT-d [76]. Furthermore, it has been suggested that female hormones may also promote BAT function in women [78,79]; however, the study did not account for the menstrual cycle. Therefore, these factors may have affected gender differences in BAT-d.

It is well known that the autonomic nervous system is predominantly regulated by reduced parasympathetic activity until the exercise intensity reaches 30–50% VO_2max_ [80,81]. Then, SNS activity begins to elevate at approximately 50% VO_2max_ and progressively increases from around 70% VO_2max_ [43,44,45,46,47]. The classification of physical activity intensity in the international physical activity questionnaire (IPAQ) in this study is W: 3.3 METs, M: 3 METs to 6 METs or less, VPA: 8 METs or higher (MET, metabolic equivalent). According to the reference value for Japanese men by the Ministry of Health, Labor and Welfare, the level of 8 METs corresponds to approximately 80% VO_2max_ in participants of the average age in this study (41.3 years) [61]. Thus, the intensity of VPA categorized in this study is considered sufficient to activate SNS [47]. We failed to find a significant relationship between BAT-d and the time spent performing VPA and between BAT-d and the frequency each week performing VPA, indicating that duration and frequency are not factors for enhancing BAT-d in this study.

The following are the limitations of this research. First, BAT-d was evaluated noninvasively using NIR_TRS_. Although NIR_TRS_ has been used for evaluating BAT-d in previous studies [39], comparisons of NIR_TRS_ to ^18^FDG-PET/CT are limited, and further research is required. NIR_TRS_ could be capable of distinguishing BAT from muscle tissue. In a study [82], tissue optical characteristics are reported to be different for deltoid muscle (μ’_s_ = 9.6 (9.1, 10.4) (medians (the first quartile, the third quartile] cm^−1^)), (total-Hb) = 114.9 (107.0, 127.7) μM) and for the supraclavicular region (μ’_s_ = 7.9 (7.2, 8.7) cm^−1^, (total-Hb) = 60.7 (48.9, 74.7) μM,). The values in the supraclavicular region in this study (μ’_s_ = 8.3 (7.3, 9.4) cm^−1^, (total-Hb) = 65.1 (50.4, 85.0) μM) are comparable to those reported earlier [82]. However, there is no concrete evidence from human studies to support the claim that NIR_TRS_ only measures BAT characteristics in the supraclavicular region. Although we attempted to avoid a large muscle such as sternocleidomastoid muscle using an ultrasound guidance, the supraclavicular region comprises other thin muscles such as the omohyoid muscle. Thus, we could not exclude a possibility that NIR_TRS_ measured not only BAT but also slightly thin muscles in this study, which, we believe, had a minor impact on the measurements. In a future study, it is necessary to compare data obtained from tissue biopsy and NIR_TRS_ measurement in humans to improve data accuracy. Although previous studies have reported no association between [Total-Hb] in the deltoid muscle region (negative control) and BAT activity measured using FDG-PET/CT [39], we have not measured [Total-Hb] in the deltoid muscle region (negative control) in this study.

The [Total-Hb] in the supraclavicular region is an index of BAT microvascular density rather than that of BAT metabolic activity. As it is well known that the [Total-Hb] in the supraclavicular region does not change during a 2-h cold exposure [39], we did not regulate the measurement time of the day and eating status. However, the confounding factors such as aging and cold stimulation, in addition to the effect of the measurement time of the day and eating status along with dietary patterns of the individuals and the consumption of spicy foods, on BAT-d, should be confirmed to improve the data accuracy in future studies.

Second, the physical activity of participants was evaluated using the international physical activity questionnaire (IPAQ). The VPA determined using IPAQ is only 8 METs or higher. According to IPAQ, VPA includes activities such as aerobics, running, fast bicycling, or fast swimming. However, it was not possible to accurately calculate the physical activity intensity or specify the type of exercise. Moreover, because IPAQ evaluates physical activity for longer than 10 minutes, it may not be possible to include a short bout (less than 10 minutes) of high intensity physical activity. Also, in the IPAQ evaluation, there may be a response bias, such as selecting a socially desirable response. Since this study does not assess the indicators of SNS activity, for instance neural activity and blood catecholamine levels, it cannot be concluded whether VPA affects BAT-d via SNS activation. In the future, it is necessary to conduct exercise training interventions that control participant characteristics and exercise mode, intensity, duration, and frequency.

Finally, since we did not measure blood myokines, which may potentially be associated with the increase in BAT-d, we would like to investigate this aspect in future studies. It would be of interest to investigate the relationship between BAT-d and thoracic visceral fat area (epicardial or pericardial fat deposits) in the future following validation of the thoracic visceral fat area using the impedance methods.

## 5. Conclusions

We conclude that vigorous-intensity physical activities are associated with high BAT-d in humans, especially in men. Furthermore, we confirmed that BAT-d in men is related to visceral fat area and VPA. In the future, we need to investigate the effects of longitudinal high-intensity exercise training on BAT-d by adjusting the exercise mode, intensity, frequency, and duration, as well as the characteristics of the participants.

## Figures and Tables

**Table 1:** Group comparisons for each parameter in men and women.

All	WM (*n* = 155)	VWM (*n* = 77)	*p*-Value
[Total-Hb] (μM)	66.6 ± 20.5	75.3 ± 26.1	<0.05
Age (years)	40.8 ± 11.7	42.2 ± 13.6	0.40
Height (cm)	161.9 ± 8.2	166.0 ± 8.4	<0.01
Weight (kg)	58.7 ± 11.0	62.0 ± 10.6	<0.05
BMI (kg/m^2^)	22.3 ± 3.1	22.4 ± 2.5	0.85
Skeletal muscle mass (kg)	23.5 ± 5.0	27.0 ± 6.0	<0.01
Skeletal muscle percentage (%)	40.1 ± 4.2	43.3 ± 4.8	<0.01
Body fat mass (kg)	15.7 ± 5.9	13.5 ± 4.8	<0.01
Fat percentage (%)	26.5 ± 7.0	22.0 ± 7.3	<0.01
Waist circumference (cm)	43.0 ± 8.0	48.5 ± 9.8	<0.01
Visceral fat area (cm^2^)	79.3 ± 8.9	79.9 ± 7.3	0.60
Fat-free mass (kg)	61.1 ± 37.6	62.1 ± 37.2	0.86
Body temperature (°C)	36.3 ± 0.4	36.3 ± 0.4	0.50
Heart rate (bpm)	71.7 ± 9.7	69.4 ± 11.9	0.14
Systolic blood pressure (mmHg)	111.7 ± 14.7	116.7 ± 14.9	<0.05
Diastolic blood pressure (mmHg)	71.0 ± 11.2	73.3 ± 10.3	0.14
Energy expenditure by physical activity (kcal/week) ^1^	1950 (1231–3651)	4420 (2183–7883)	<0.01
Physical activity time at vigorous-intensity (min/week) ^1^	0	180 (60–360)	<0.01
Physical activity time at moderate-intensity min/week) ^1^	210 (100–510)	180 (93–470)	0.49
Physical activity time at intensity of equal to walking (min/week) ^1^	250 (120–480)	350 (188–660)	<0.05

Results are presented as the mean ± standard deviation. An independent *t*-test was performed to determine the significance of the group differences. ^1^ Indicated by the median according to guidelines [60]; the Mann–Whitney test was used to determine the significance of group differences. VWM—a group performing all physical activities including walking, moderate physical activity, and vigorous-intensity physical activity; WM—a group performing physical activities including walking and moderate physical activity; [Total-Hb]—total hemoglobin concentration in the supraclavicular region adjusted according to the thickness of the subcutaneous adipose tissue layer; BMI—body mass index.

**Table 2:** Group comparison of each parameter in women.

Women	WM (*n* = 109)	VWM (*n* = 36)	*p*-Value
[Total-Hb] (μM)	68.1 ± 19.9	70.9 ± 21.8	0.48
Age (years)	41.2 ± 12.8	45.4 ± 15.7	0.15
Height (cm)	158.2 ± 5.9	158.9 ± 5.4	0.51
Weight (kg)	54.2 ± 8.1	53.3 ± 4.5	0.42
BMI (kg/m^2^)	21.6 ± 2.9	21.1 ± 1.8	0.21
Skeletal muscle mass (kg)	20.8 ± 2.3	21.5 ± 2.8	0.10
Skeletal muscle percentage (%)	38.7 ± 3.6	40.4 ± 4.0	<0.05
Body fat mass (kg)	15.6 ± 5.7	13.9 ± 3.6	<0.05
Fat percentage (%)	28.2 ± 6.5	26.0 ± 6.0	0.08
Waist circumference (cm)	38.5 ± 3.9	39.4 ± 4.2	0.26
Visceral fat area (cm^2^)	76.7 ± 8.0	76.0 ± 5.9	0.59
Fat-free mass (kg)	47.7 ± 26.7	42.4 ± 18.5	0.27
Body temperature (°C)	36.3 ± 0.4	36.3 ± 0.4	0.35
Heart rate (bpm)	72.2 ± 9.4	68.5 ± 10.7	0.06
Systolic blood pressure (mmHg)	108.3 ± 14.4	112.8 ± 16.9	0.13
Diastolic blood pressure (mmHg)	68.2 ± 10.9	70.4 ± 10.1	0.30
Energy expenditure by physical activity (kcal/week) ^1^	2073 (1292–3725)	4288 (2086–7052)	<0.01
Physical activity time at vigorous-intensity (min/week) ^1^	0	160 (60–345)	<0.01
Physical activity time at moderate-intensity min/week) ^1^	240 (95–630)	255 (120–638)	0.49
Physical activity time at intensity of equal to walking (min/week) ^1^	280 (128–555)	350 (165–743)	<0.05

Results are presented as the mean ± standard deviation. An independent *t*-test was performed to determine the significance of the group differences. ^1^ Indicated by the median according to guidelines [60]; the Mann–Whitney test was used to determine the significance of group differences. VWM—a group performing all physical activities including walking, moderate physical activity, and vigorous-intensity physical activity; WM—a group performing physical activities including walking and moderate physical activity; [Total-Hb]—total hemoglobin concentration in the supraclavicular region adjusted according to the thickness of the subcutaneous adipose tissue layer; BMI—body mass index.

**Table 3:** Group comparison of each parameter in men.

Men	WM (*n* = 46)	VWM (*n* = 41)	*p*-Value
[Total-Hb] (μM)	63.3 ±21.8	79.2 ±29.1	< 0.01
Age (years)	39.9 ±8.6	39.4 ±10.9	0.83
Height (cm)	170.8 ±5.5	172.3 ±4.8	0.18
Weight (kg)	69.5 ±9.2	69.7 ±8.2	0.92
BMI (kg/m^2^)	23.8 ±3.1	23.4 ±2.5	0.53
Skeletal muscle mass (kg)	30.0 ±3.1	31.8 ±3.1	<0.01
Skeletal muscle percentage (%)	43.5 ±3.7	45.9 ±3.9	<0.01
Body fat mass (kg)	16.0 ±6.3	13.2 ±5.7	<0.05
Fat percentage (%)	22.5 ±6.4	18.5 ±6.6	<0.01
Waist circumference (cm)	53.5 ±5.0	56.5 ±5.1	0.83
Visceral fat area (cm^2^)	85.5 ±7.8	83.3 ±6.8	0.18
Fat-free mass (kg)	93.5 ±40.6	79.3 ±40.9	0.92
Body temperature (°C)	36.2 ±0.4	36.3 ±0.4	0.53
Heart rate (bpm)	70.5 ±10.4	70.1 ±12.9	<0.01
Systolic blood pressure (mmHg)	120.1 ±11.6	120.2 ±12.0	<0.01
Diastolic blood pressure (mmHg)	77.8 ±9.1	75.9 ±9.8	<0.05
Energy expenditure by physical activity (kcal/week) ^1^	1806 (1061–3539)	4876 (2530–8629)	<0.01
Physical activity time at vigorous-intensity (min/week) ^1^	0	180 (60–360)	<0.01
Physical activity time at moderate-intensity min/week) ^1^	155 (93–308)	130 (60–300)	0.93
Physical activity time at intensity of equal to walking (min/week) ^1^	213 (104–360)	340 (200–585)	<0.01

Results are presented as the mean ± standard deviation. An independent *t*-test was performed to determine the significance of the group differences. ^1^ Indicated by the median according to guidelines [60]; the Mann–Whitney test was used to determine the significance of group differences. VWM, a group performing all physical activities including walking, moderate physical activity, and vigorous-intensity physical activity; WM, a group performing physical activities including walking and moderate physical activity; [Total-Hb], total hemoglobin concentration in the supraclavicular region adjusted according to the thickness of the subcutaneous adipose tissue layer; BMI, body mass index.

**Table 4:** Multiple regression analysis with [Total-Hb] in the supraclavicular region as an independent variable.

[Total-Hb]	Univariate Regression		Multivariate Regression	
All	r	*p*	Standardized β	*p*
Age (years)	−0.07	0.16	-	-
BF (%)	−0.50	<0.01	−0.39	<0.01
VFA (cm^2^)	−0.46	<0.01	−0.33	<0.01
WM-VWM	0.18	<0.01	-	-
			R^2^ = 0.34	
Women				
Age (years)	−0.03	0.34	-	-
BF (%)	−0.47	<0.01	−0.47	<0.01
VFA (cm^2^)	−0.44	<0.01	-	-
WM-VWM	0.06	0.24	-	-
			R^2^ = 0.22	
Men				
Age (years)	−0.12	0.13	-	-
BF (%)	−0.64	<0.01	-	-
VFA (cm^2^)	−0.66	<0.01	−0.62	<0.01
WM-VWM	0.32	<0.01	0.21	<0.05
			R^2^ = 0.47	

The categorical variables were set at “0” for the group that only walked and performed moderate-intensity physical activity (WM) and “1” for the group that performed all intensity physical activities (walking, moderate, and vigorous intensity—VWM). Abbreviations: BF—body fat; VFA—visceral fat area.
